# Clinical efficiency of bone marrow mesenchymal stem cell implantation for osteonecrosis of the femoral head: a matched pair control study with simple core decompression

**DOI:** 10.1186/s13287-018-1030-y

**Published:** 2018-10-25

**Authors:** Joon Soon Kang, Young Ju Suh, Kyoung Ho Moon, Jun Sung Park, Tae Hoon Roh, Myung Hoon Park, Dong Jin Ryu

**Affiliations:** 1 Department of Orthopedic Surgery, College of Medicine, Inha University Hospital, 7-206, 3rd Street Sinheung-Dong, Jung-Gu, Incheon, 400-103 South Korea; 2 Department of Biomedical Sciences, College of Medicine, Inha University, Incheon, South Korea

**Keywords:** Femur head, Osteonecrosis, Mesenchymal stem cell, Core decompression, AVN

## Abstract

**Background:**

To date, several trials have reported the use of mesenchymal stem cell (MSC) implantation for osteonecrosis of the femoral head (ONFH). However, the clinical outcomes have not been conclusive. This study compared the clinical and radiological results of bone marrow mesenchymal stem cell (BMMSC) implantation with traditional simple core decompression (CD) using a matched pair case–control design.

**Methods:**

We retrospectively reviewed 100 patients with ONFH (106 hips) who had been treated by CD alone (50 patients, 53 hips) and CD + BMMSC implantation (50 patients, 53 hips) between February 2004 and October 2014. We assessed the total hip replacement arthroplasty (THA) conversion rate and ARCO (Association Research Circulation Osseous) stage progression. Survivor rate analysis was performed using the Kaplan–Meier method, and an additional THA was defined as the primary endpoints.

**Results:**

The mean follow-up period was 4.28 years. There was a difference in the THA conversion rate between the CD (49%) and CD + BMMSC groups (28.3%) (*p* = 0.028). ARCO stage progression was noted in 20 of 53 hips (37.7%) in the CD group and 19 of 53 hips (35.8%) in the CD + BMMSC group. Among collapsed cases (ARCO stages III and IV), there was no difference in clinical failure rate between the two groups. Conversely, in the pre-collapse cases (ARCO stages I and II), only 6 of 30 hips (20%) progressed to clinical failure in the CD + BMMSC group, whereas 15 of 30 hips (50%) progressed to clinical failure in the CD group (*p* = 0.014). Kaplan–Meier survival analysis showed a significant difference in the time to failure between the two groups up to 10-year follow-up (log-rank test *p* = 0.031). There was no significant difference in terms of age (*p* = 0.87) and gender (*p* = 0.51) when comparing THA conversion rates between groups. No complication was noted.

**Conclusions:**

These results suggest that implantation of MSCs into the femoral head at an early stage of ONFH lowers the THA conversion rate. However, ARCO stage progression is not affected by this treatment.

**Trial registration:**

Retrospectively registered

## Background

Osteonecrosis of the femoral head (ONFH) is still a challenging disease in orthopedics, frequently leading to femoral head collapse. Without effective early treatment, ONFH finally progresses to osteoarthritis which can only be treated by a total hip replacement arthroplasty (THA). Core decompression (CD) is a widely used procedure for treating ONFH, but the clinical outcomes remain controversial. In a systematic review, the total clinical success rate of CD, with or without cancellous bone transplantation, was only 63.5% and that of joint replacement surgery or hip rescue operation was approximately 33% [1]. Vascularized fibular grafting introduced more satisfactory results, especially at an early stage, but it was associated with morbidity at the donor site and adverse events.

Recent publications suggested that ONFH may be a disease involving the bone and mesenchymal cells. In ONFH patients, the number and level of MSC’s activity in the bone marrow was found to be decreased [2]. Gangji et al. reported that osteoblast replication capacity is decreased in the proximal femur of ONFH patients [3]. Additionally, the rates of osteoblast and bone cell apoptosis were found to be increased in the necrotic region [4, 5]. This finding showed that transplanting fresh mesenchymal stem cells into the necrotic lesion may be a useful treatment option. Hernigou et al. [6] introduced intra osseous injection of a high concentration of bone marrow stem cells, and the clinical outcome was better than that of CD treatment previously performed alone. After this trial, transplanting autologous bone marrow mesenchymal stem cells (BMMSC) into the core decompression pathway has become effective treatment for ONFH [7–9]. However, recently, several studies reported that there is no difference in the clinical outcomes between CD and BMMSC treatment [10–12]. Thus, the therapeutic effect of implanting BMMSC in ONFN patients is still under debate. The present study investigated the outcome of the two treatments, taking into consideration various factors which may affect the progression of avascular necrosis (AVN), such as age, gender, etiology, body mass index (BMI), and initial stage of ONFH.

Therefore, the primary aim of this study is to compare the clinical and radiological outcomes of stem cell implantation with simple CD and with CD + BMMSC using matched pair case–control design. The secondary aim is to investigate the ARCO stage and patient population produce best outcomes if BMMSC injection therapy is effective for ONFH.

## Methods

### Patients

**Fig. 1:**
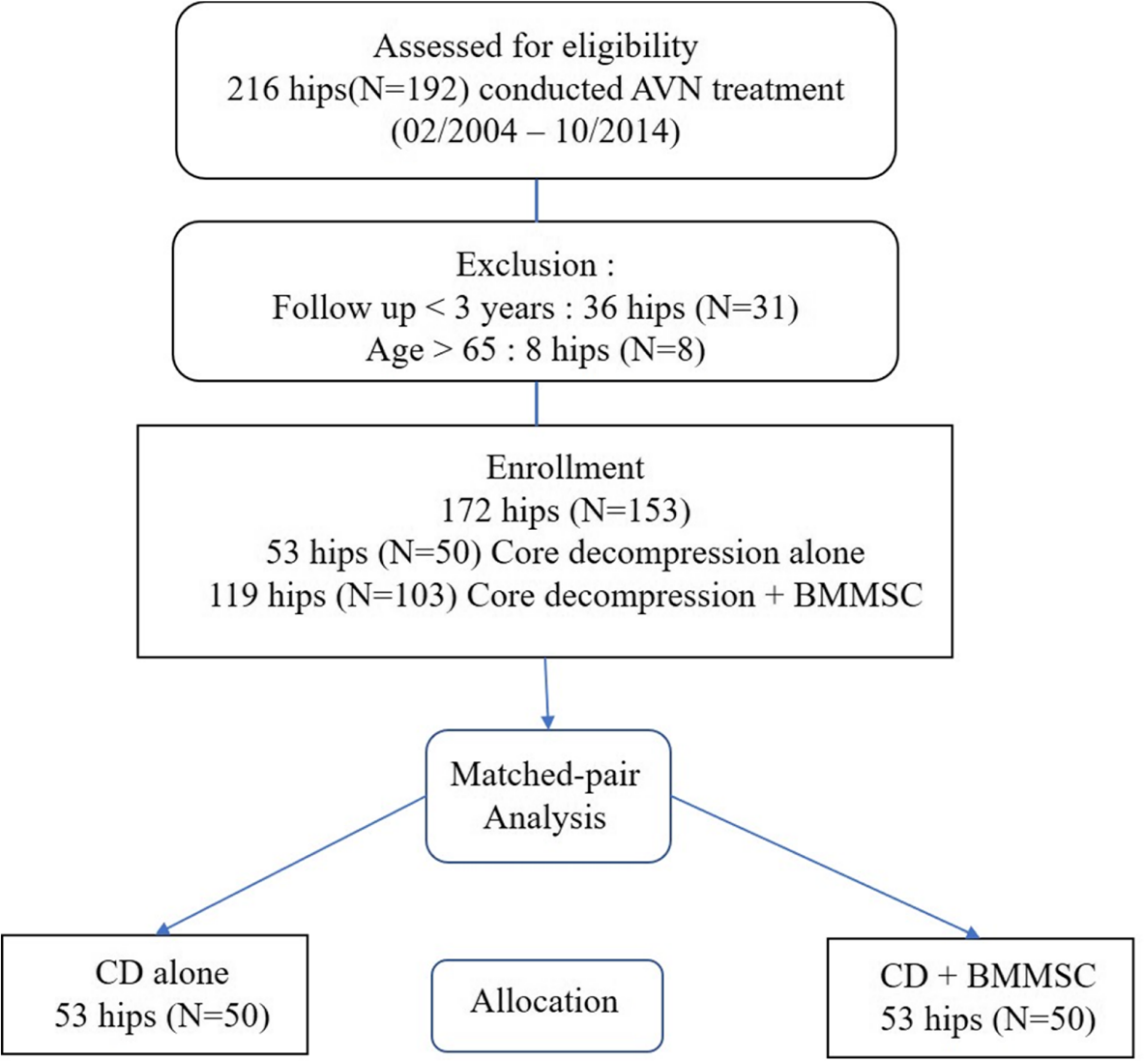
Flow chart of the patient selection and matching process. AVN avascular necrosis of femoral head, BMMSC bone marrow mesenchymal stem cell, CD core decompression

We conducted a retrospective study on 100 patients (106 hips) of ONFH, who had been treated with traditional CD alone or with CD + BMMSC transplantation between February 2004 and October 2014. The protocol of this study was approved by the Inha University Hospital Institutional Review Board (approval number: INHAUH 2018-08-008-001). The follow-up period ranged from at least 3 years to at most 10 years. A retrospective matched pairs, case–control analysis was performed. The patient selection and matching process is presented in Fig. 1. The patients were matched by age (± 3 years), gender, cause of the ONFH, BMI (± 2 kg/m^2^), initial ARCO stage, follow-up period (± 0.5 years), and surgeon. The mean age at the time of surgery was 46.7 years (22~65 years). Seventy-four patients were men, and 26 were women. Both groups consisted of 50 patients (53 hips, including 3 cases where both the hips affected). Detailed demographic data are presented in Table 1. The patient was allowed to choose between the two surgical methods after the procedures were explained and information about the expenditures was relayed. The patients for whom ONFH had been developed because of trauma, for whom MRI had not been performed, who had stopped steroid therapy, and who had undergone previous CD treatment or who had any malalignment were excluded.

**Table 1:** Demographics of group I (CD alone) and group II (CD + BMMSC)

	Group I (CD alone)	Group II (CD + BMMSC)	*p* value
Cases (hips)	50 (53)	50 (53)	n.s.
Gender (male to female)	38:12 (76%:24%)	36:14 (72%:28%)	n.s.
Age (years)	47.3 ± 9.7 (22–65)	46.0 ± 9.3 (24–64)	n.s.
Cause			n.s.
-Alcohol	19	19	
-Corticosteroid	5	5	
-Others	5	5	
-Idiopathic	24	24	
BMI	24.0 ± 4.1	23.8 ± 3.7	n.s.
VAS score	4.2 ± 1.1	4.8 ± 1.3	n.s.
ARCO stage (I:II:III:IV)	(1:29:19:4)	(1:29:19:4)	n.s.
Pre-collapse (stages I, II) to collapse (stages III, IV)	30:23	30:23	n.s.
Average follow-up period (years)	4	4.53	n.s.

*n.s* not significant, *CD* core decompression, *BMMSC* bone marrow mesenchymal stem cell, *BMI* body mass index, *VAS* visual analogue scale

### Surgical technique

#### CD treatment

All patients underwent surgery under spinal anesthesia. Guided by a fluoroscopic view through the greater trochanter and the femoral neck, using 2.0-mm K-wires, core decompression tunnels were made into the subchondral necrotic lesion of the femoral head (2–3 mm away from the joint cartilage). The centrally positioned K-wire was over drilled using trephine. Subsequently, the necrotic tissues were removed from the femoral head.

#### CD + BMMSC treatment

The CD + BMMSC procedure was identical to the CD treatment regarding the procedure of the decompression tunnel creation and the removal of the necrotic lesion of the femoral head, as described above. The bone marrow was aspirated up to 100–120 mL through the decompression tunnel at the subtrochanteric, proximal femur area, and diluted with the same amounts of Hanks’ balanced salt solution (HBSS; GIBCO-BRL, Grand Island, NY, USA). Then, Ficoll-Paque Plus (1077 g/L; Amersham Biosciences, Piscataway, NJ, USA) solution was added in the same ratio. Centrifugation for 30 min at 1000*g* separated the mononuclear cells from the remainder of the marrow. The mononuclear cells were then collected and washed with HBSS three times before another 15-min centrifugation at 900*g*. Subsequently, the mononucleocyte layer was collected and 1.8 mL of phosphate buffered saline (Gibco, Carlsbad, CA, USA) was added to suspend the cells. The mean injected leukocyte cell count and average stem cells per colony-forming units (CFUs) were not calculated for all patients. However, in 17 hips in 16 patients, the cell counts were confirmed by a pilot study. The average density of the autologous marrow monocytes was 13.97 ± 13.8 × 10^6^/mL (range from 1.37 × 10^6^/mL to 59.6 × 10^6^/mL). The mean number of fibroblast CFUs per million nucleated cells obtained from each patient was 21.6 ± 11.01 (range from 7 to 53). The number of collected cells was slightly lower than that reported in previous studies [3, 13], but it was not particularly lower than that reported in other stem cell injection studies [6, 8, 14] So, it was considered that there would be enough cell count in obtaining the therapeutic effect. About 15 mL of autologous marrow monocyte solution was injected into the necrotic lesion by a syringe. To avoid leakage, the decompression tunnel was plugged by bone core. Subsequently, the decompression tunnel was sealed using bone wax. All surgical procedures were performed by two senior hip surgeons (KH Moon and JS Kang).

### Clinical evaluation

ONFH was diagnosed using X-ray and MRI [15]. ONFH was classified into different stages according to the ARCO classification system [16]. We surveyed the groups that had undergone CD alone and CD + BMMSC treatment. We analyzed THA conversion rate, the ARCO stage deterioration degree, and the last f/u visual analogue scale for pain (VAS) score. The patients were divided into alcohol, steroid, other (aplastic anemia, sickle cell disease, etc.), and idiopathic groups according to the cause of condition. Additionally, to evaluate the difference in disease progression, we divided the patients into pre-collapse stage (ARCO stages I and II) and collapsed stage (ARCO stages III and IV). We defined the primary endpoint as the need for additional surgery (e.g., THA conversion); THA conversion and VAS score were recorded for clinical outcome evaluation. Preoperative ARCO stage classification was evaluated with anteroposterior and lateral radiographs and MRI. Follow-up radiographic and clinical examinations were performed at 6 weeks, 3 months, 6 months, 1 year, and annually thereafter. Follow-up radiographs were used to assess the progression of ARCO stage [11, 17–19].

### Statistical analysis

We used chi-square test and Fisher’s exact test to compare the differences in clinical and radiological outcome between the two groups. Survivor rate analysis was conducted using the Kaplan–Meier method. Additional THA surgery was defined as the primary endpoints. We used log-rank test to calculate the statistical significance of the differences between the Kaplan–Meier survival curves of the two groups. A *p* < 0.05 was considered statistically significant. Statistical analyses were performed using the SPSS software (ver. 18.0; SPSS, Inc., Chicago, IL, USA).

## Results

**Fig. 2:**
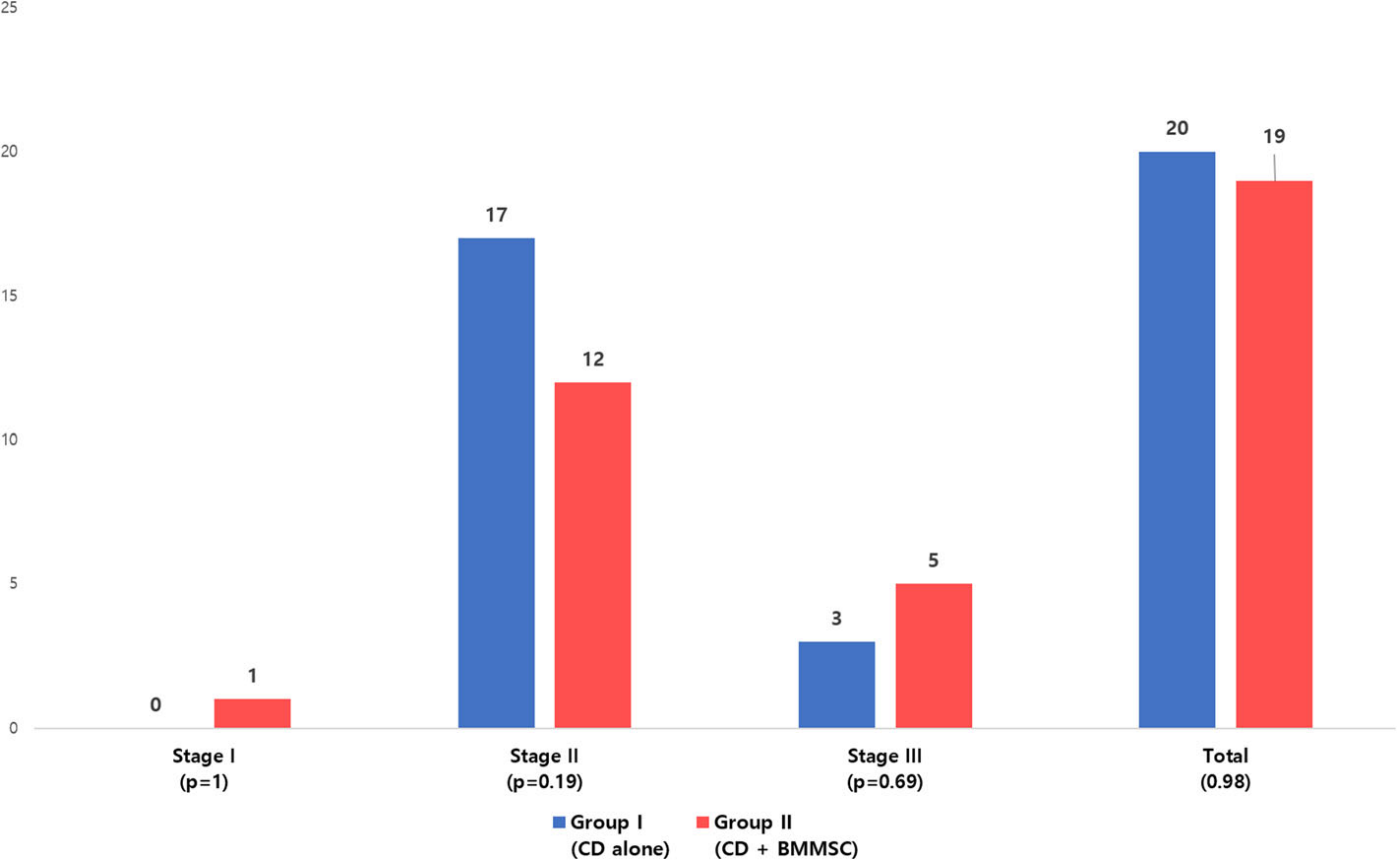
Cases of ARCO stage progression in each group

**Fig. 3:**
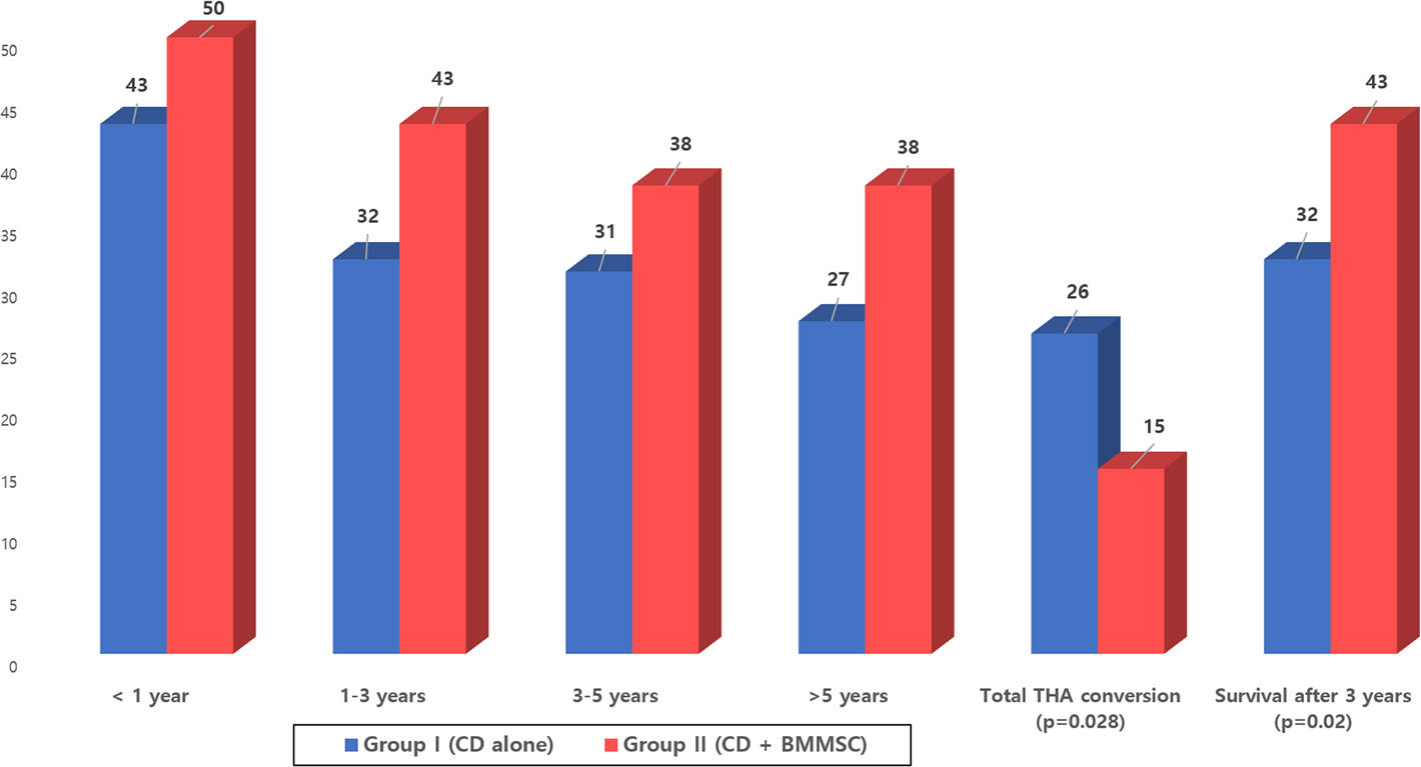
Survival of hips over time follow-up

**Fig. 4:**
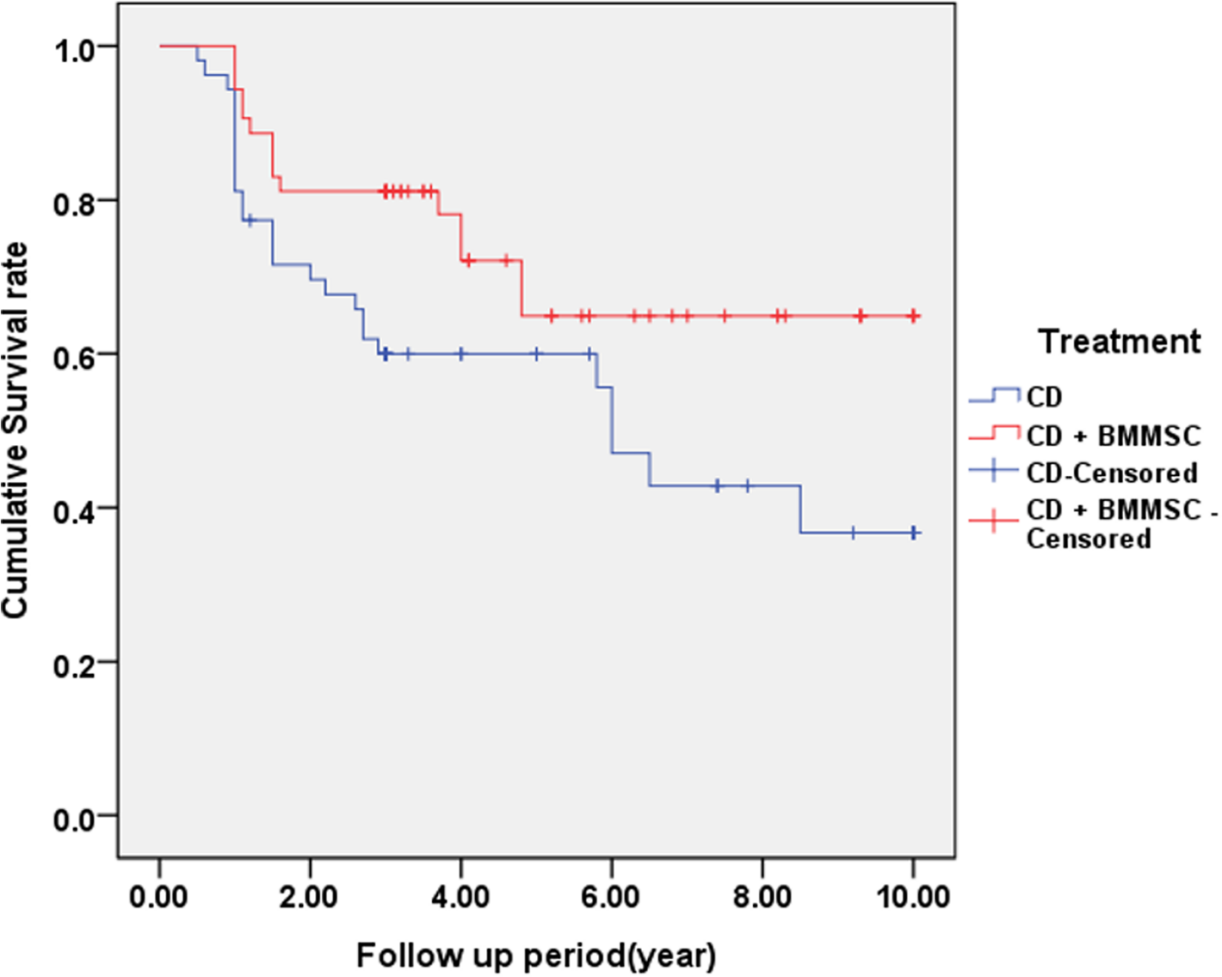
Femoral head survival rates of CD and CD + BMMSC groups according to the Kaplan–Meier method. The difference between the two groups was statistically significant (log-rank test, *p* = 0.031)

**Fig. 5:**
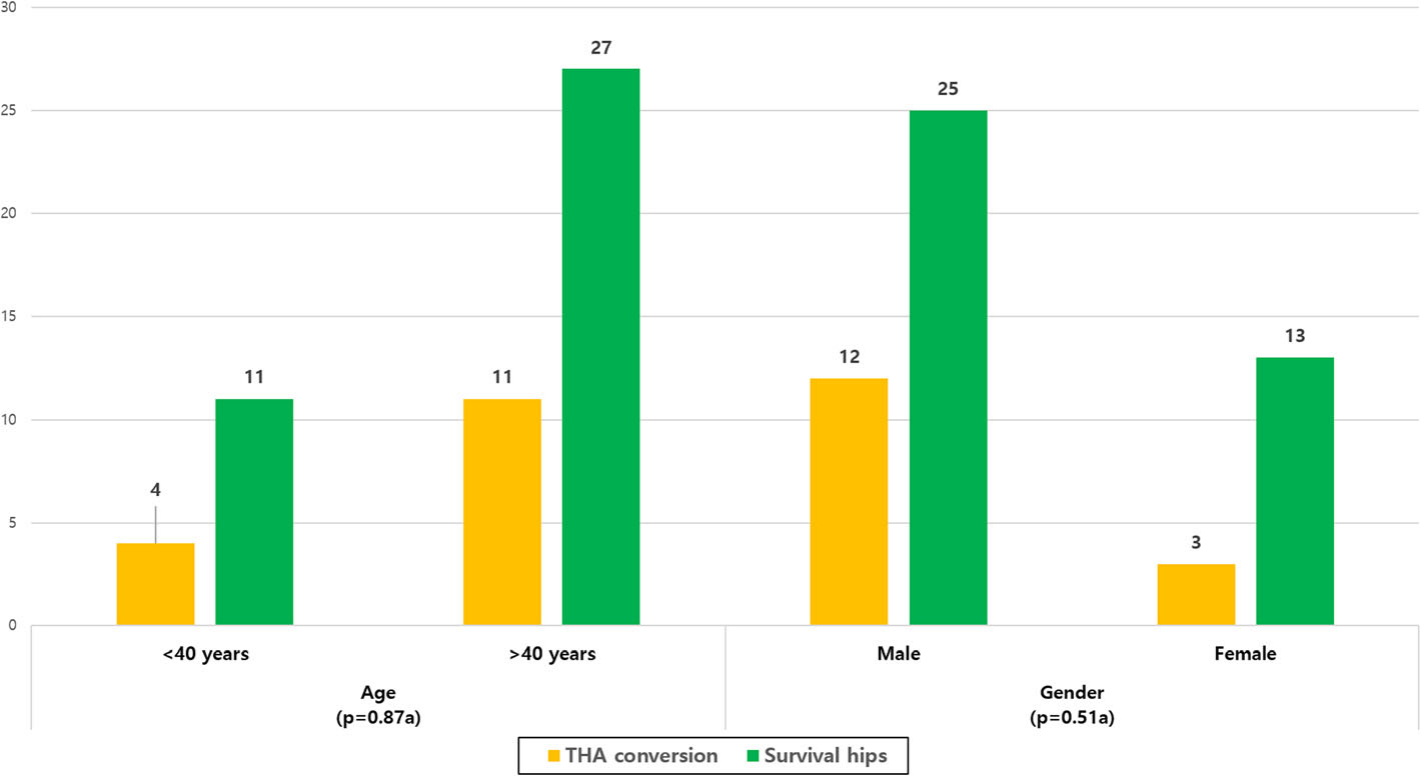
THA conversion ratio based on age and gender in CD + BMMSC group

There was no noted case of related complications such as malignancy, bone overgrowth, fracture along the core track, perforations in the femoral head, deep vein thrombosis (DVT), and infection during the respective follow-up periods (3–10 years). Based on the matched pair, case–control study design, no significant differences were found between the two groups in terms of age, gender, cause of the ONFH, BMI, ARCO stage, and follow-up period (Table 1). The mean follow-up period was 4.28 years. In the CD alone group, 20 hips (40.8%) demonstrated ARCO stage progression at the last follow-up. Twenty-six of 53 hips (49%) required additional surgery (THA). However, in the CD + BMMSC group, 19 hips (38.8%) demonstrated ARCO stage progression at the last follow-up. Fifteen hips (28.3%) required THA surgery (Figs. 2 and 3). We observed statistically significant difference (*p* = 0.031) in the femoral head survival (THA conversion) rate between the CD and CD + BMMSC groups using Kaplan–Meier survivorship curves at the 10-year time point (Fig. 4). The CD + BMMSC group showed higher (43 of 53 hips) femoral head survival rate especially for more than 3 years than the CD alone group (32 of 53 hips). This difference was statistically significant (*p* = 0.02) (Fig. 3). There was no significant difference in the THA conversion rate based on age (*p* = 0.87) and gender (*p* = 0.51) in the CD + BMMSC group (Fig. 5).

**Fig. 6:**
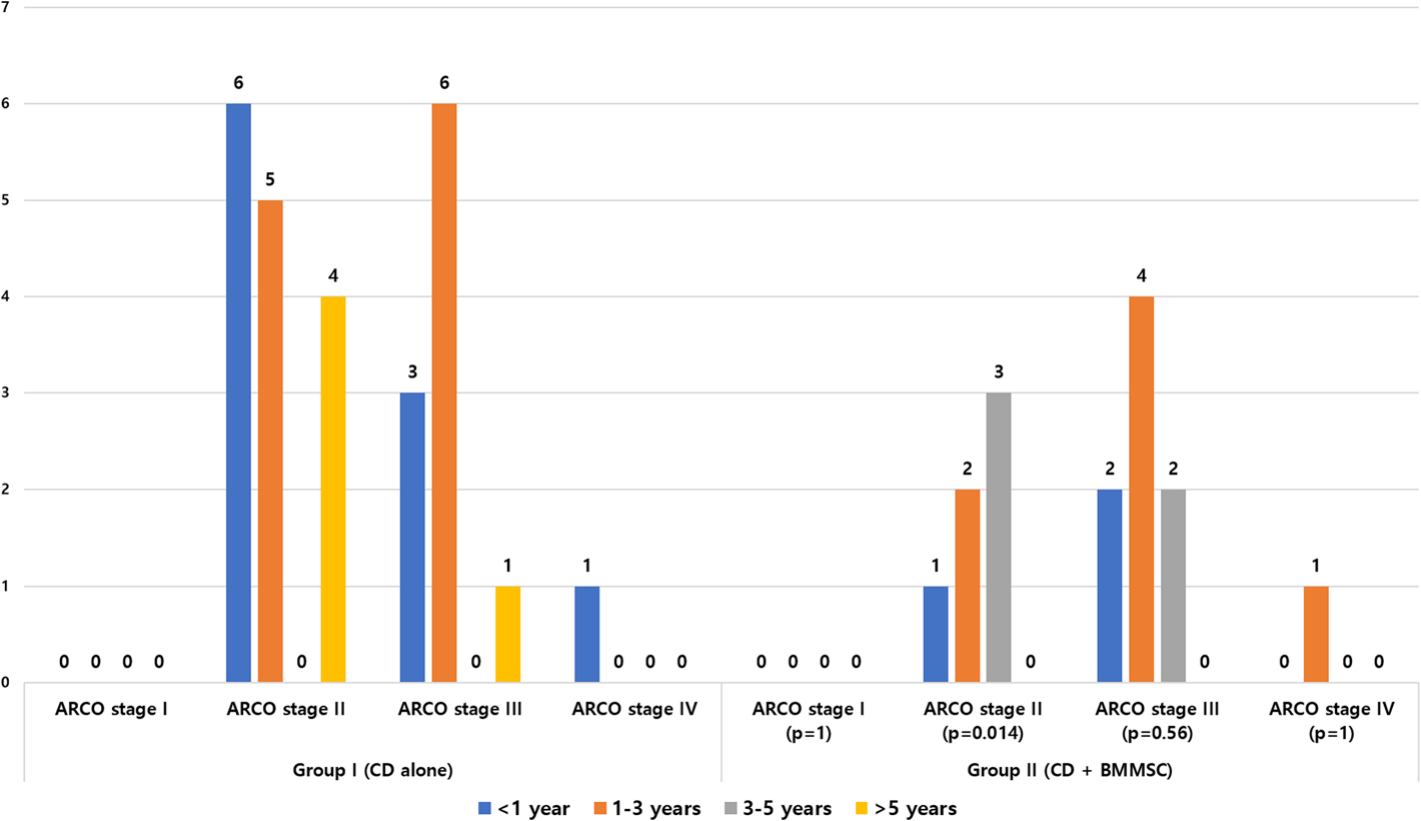
THA conversion cases for each group according to ARCO stage

In early-stage (pre-collapse) patients, there was significant difference in THA conversion rate between two groups (*p* = 0.014). In the CD group, 15 hips needed THA conversion (50%) in the pre-collapse stage. However, in the CD + BMMSC group, there were only 6 cases that needed THA conversion (16.7%) (Fig. 6). However, there was no difference in ARCO stage progression at each stage (Fig. 2) between the two groups. At the last follow-up, the mean VAS score was 2.1 for the CD group and 2.3 for the CD + BMMSC group. This difference was not statistically significant (*p* = 0.450).

## Discussion

In this study, CD + BMMSC implantation was found to be an effective method for reducing THA conversion rate in ONFH patients. Especially in early-stage (pre-collapse) patients, CD + BMMSC implantation reduces THA conversion rate (6 cases of 30 hips), but it could not prevent ARCO stage progression. Early stage at treatment (ARCO stages I and II) is the only important factor which affects the reduction of the THA conversion rate. These results were obtained after excluding some factors which could affect the clinical and radiological outcomes, and this is an advantage of the current study.

Most previous studies have evaluated the outcomes considering THA conversion rate, and ARCO/Ficat stage progression or worsening of the hip score. However, there are many patients who do not undergo additional THA surgery because of fear for surgery, social economic status, psychological factor (fear for losing of their own joint), religious belief, etc. [20, 21]. Furthermore, there are some patients who complain of pain aggravation or functional disability without ARCO stage progression. On the other hand, despite ARCO stage progression or collapsing femoral head, there are some patients who do not experience aggravation of pain or functional disability. Osteonecrosis was found to be the main reason for conversion to total hip arthroplasty, but not all patients with osteonecrosis required further surgery [22]. We defined the primary endpoint as the need for additional surgery (e.g., THA conversion) to evaluate the clinical outcome. Also to evaluate the radiological outcome, we assessed the ARCO stage progression. However, these two outcomes could not be correlated because of the above reason. Although not statistically significant in this study, the initial VAS score was higher in the CD + BMMSC group. This may be a result of patients with severe pain or in a serious condition selecting stem cell therapy, which is thought to be more effective. This in turn may have affected the postoperative satisfaction and pain scores. As mentioned above, factors such as psychological factors and personal condition which are difficult to quantify may have influenced the clinical outcome.

BMMSCs have the potential to differentiate into osteoblasts, endothelial progenitor cells, and hemangioblasts, all of which function to repair the necrotic region. Although BMMSC implantation technique is effective for early-stage ONFH as demonstrated in the current and previous studies [8, 23–25], it is not effective for the collapsed stage [26]. As ONFH mainly affects young and middle-aged adults and THA cannot be expected to last for the patient’s lifetime, hip-preserving treatments are especially important for these patients [20, 21]. Therefore, further studies are needed for effective and safe stem cell therapy methods for the young and late-stage patients. In an experimental study by Lavasani et al. [27], the authors suggested that the therapeutic effects of the MSCs might be mediated by secreted factors. However, the mechanisms by which MSCs potentially act remain the subject of further investigation. Recently, vascular endothelial cell growth factor (VEGF) combined with bone morphogenetic protein (BMP) was used to repair avascular necrosis of the femoral head [28]. This method can maintain the osteogenic phenotype of seed cells and effectively promote blood vessel regeneration and contribute to formation and revascularization of engineered bone tissues.

This study has several limitations. First, this was a retrospective study. Second, the patients chose the surgical procedure. This complies with ethical issues, but it can lower the power of the study. Third, we retrospectively reviewed the records from only one hospital, which affected selection bias. Fourth, after a minimum 3-year follow-up, there were some patients who were lost to follow-up, which would affect the ARCO stage and pain scores and the THA conversion rate. Fifth, complete data on the exact number of injected mononuclear cells was not available. Only cell counts for 17 hips in 16 patient data were collected, and this does not represent the actual injected cell counts for all patients. The number of collected mononuclear cells may vary from patient to patient, and the differentiation potential of cells may be different [29, 30]. These are likely to have had a significant impact on the outcome. Furthermore, there was no data on cell surface markers. Therefore, we could not clarify the qualitative result and this is a weak point of our study. Sixth, bone marrow aspiration was conducted in the subtrochanteric and proximal femur area. This method has an advantage that it does not require an additional incision for bone marrow harvesting and is effective for reducing pain, which is the most common postoperative complication [8, 14, 31], thus reducing morbidity and improving patient satisfaction. However, for the bone marrow collected from this area, the number of mononuclear cells may be insufficient, and the differentiation potential may also be relatively decreased, especially in advanced lesions such as stages III and IV. For stem cell therapy, the number and differentiation potential are quite important factors because they can determine the success or failure of the therapy. The average mononuclear cell count and fibroblast-CFU determined in this study were generally lower than those reported in few previous studies [32, 33]. However, they were similar to or even higher than the lowest levels of other studies reporting successful treatment outcomes [8, 17, 34]. In general, cells obtained from older patients or those with advanced lesions are known to have lower differentiation levels. However, there were some reports suggesting that sufficient numbers of MSCs can be collected from the old age patients, patients with end-stage arthritis, proximal femur, and distal femur. Further, the differentiation potential in the abovementioned cases has been confirmed [14, 29, 35]. In addition, mononuclear stem cells from young-aged healthy donors have been reported to differ between donors in their differentiation potentials [30]. Other factors such as comorbidities and medication can affect the quality of bone marrow aspirates [36]. There is still a lack of standardized consensus about the quantitative and qualitative cell count, methods for cell harvest, cell processing, and cell transplantation/delivery. A large number of blinded randomized control trials and clinical effectiveness trials are required to clarify the treatment standard.

Despite these limitations, our study has some merits. A matched pair study design was used to improve the reliability of the results by matching for age, sex, BMI, ARCO stages, and cause of ONFH as these could affect the outcome. In our study, BMMSCs were isolated from all patients; therefore, the study is free from ethical issues. No direct complications such as excessive new bone formation and malignancy were observed in this study. Moreover, no complications have been reported from this method [37]. Further, long-term outcomes were considered through a follow-up period of up to 10 years.

## Conclusion

This study suggests that implantation of autologous MSCs into the femoral head in the early stage of ONFH lowers the THR conversion rate. However, the progression of the ARCO stage is not affected by this treatment.

## Abbreviations

MSC: Mesenchymal stem cell
ONFH: Osteonecrosis of the femoral head
CD: Core decompression
BMMSC: Bone marrow mesenchymal stem cell
THA: Total hip arthroplasty
ARCO: Association Research Circulation Osseous
AVN: Avascular necrosis
BMI: Body mass index
VAS: Visual analogue scale
n.s: Not significant
